# Effective and Reliable Framework for Lung Nodules Detection from CT Scan Images

**DOI:** 10.1038/s41598-019-41510-9

**Published:** 2019-03-21

**Authors:** Sajid Ali Khan, Shariq Hussain, Shunkun Yang, Khalid Iqbal

**Affiliations:** 1 Department of Software Engineering, Foundation University Islamabad, Islamabad, Pakistan; 2 Department of Computer Science, Shaheed Zulfikar Ali Bhutto Institute of Science and Technology, Islamabad, Pakistan; 3 School of Reliability and Systems Engineering, Beihang University, Beijing, China; 4 COMSATS University Islamabad, Attock Campus, Attock, Pakistan

## Abstract

Lung cancer is considered more serious among other prevailing cancer types. One of the reasons for it is that it is usually not diagnosed until it has spread and by that time it becomes very difficult to treat. Early detection of lung cancer can significantly increase the chances of survival of a cancer patient. An effective nodule detection system can play a key role in early detection of lung cancer thus increasing the chances of successful treatment. In this research work, we have proposed a novel classification framework for nodule classification. The framework consists of multiple phases that include image contrast enhancement, segmentation, optimal feature extraction, followed by employment of these features for training and testing of Support Vector Machine. We have empirically tested the efficacy of our technique by utilizing the well-known Lung Image Consortium Database (LIDC) dataset. The empirical results suggest that the technique is highly effective for reducing the false positive rates. We were able to receive an impressive sensitivity rate of 97.45%.

## Introduction

Lung cancer is the world’s most common cancer in terms of patients and deaths every year. One of the reasons for greater number of deaths caused by lung cancer is the inability to diagnose lung cancer in the early stages as the symptoms tend to appear in the later stages^1^. Computer aided diagnosis of Lung CT images is considered an effective technique for the detection of lung abnormally nodules. The nodules can have a variety of causes e.g. infections, sarcoidosis, Hamartoma, Wegener’s granulomatosis, Pneumoconiosis, Tuberculosis, hypersensitivity Pneumonia and Cancer. The detected nodules are located in the lung region of the CT scan. Such area is normally less than half the area of the CT slice. It takes considerably longer time to locate or search for a nodule in the whole slice. In order to reduce this complexity, it is better to reduce the search space by considering only that part of the slice where the nodule may exist. This demands a technique for segmenting the part of the lungs. In this research work, we propose a fully automated segmentation technique based on Genetic and image processing techniques based on morphology. The proposed technique would serve as a pre-processing step of CAD and will greatly benefit the nodule detection process.

**Figure 1:**
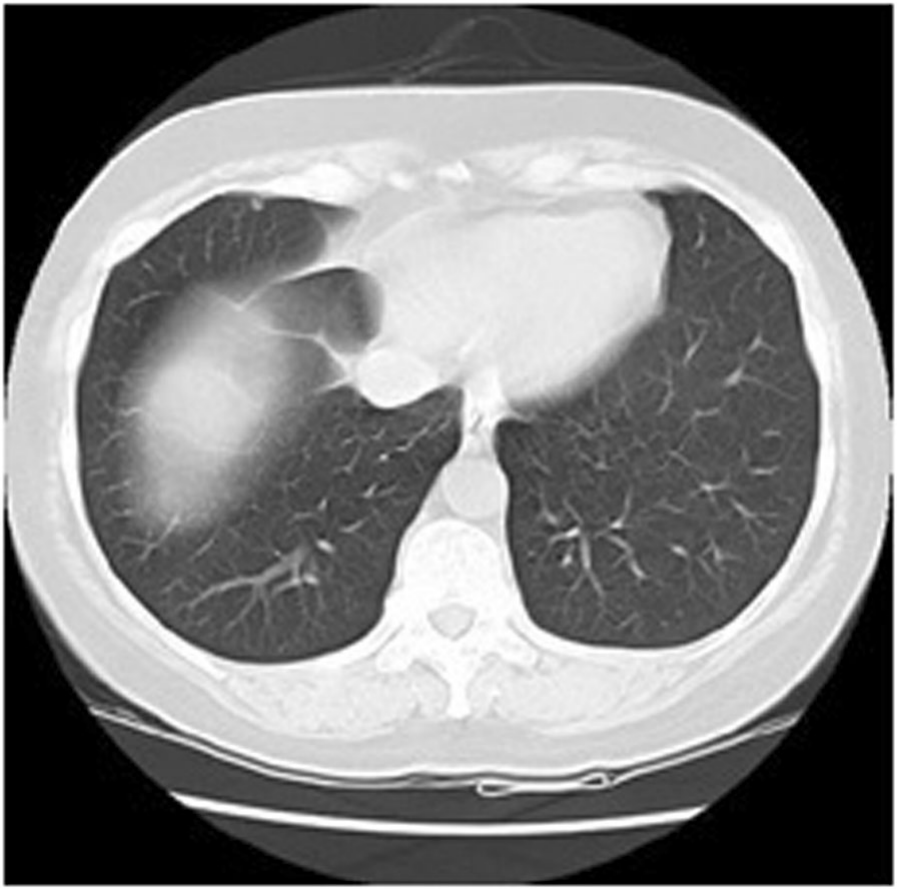
Original Lungs CT scan image.

Segmentation can be defined as a process that partitions a digital image into different set of segments. The Segmentation process transforms an image into a more meaningful form. Analysis of resultant segments is also comparatively easier ^2^. The resultant set of segments collectively covers the entire image. The pixels in each region have some similar characteristics like intensity, colour, or texture although characteristics wise, the adjacent regions tend to be significantly different. Figure 1 illustrate the sample lungs CT scan image.

Many researchers have proposed different lung segmentation algorithms and authors in^3^ provide a comprehensive review of these algorithms. The proposed methods can be classified into three basic categories^4^.

### Simple

This class of algorithms include low complexity techniques like region growing^5,6^ and their basis are formed on simplistic assumptions (e.g., density range of lung tissue). These techniques have a major advantage of being computational inexpensive and provide efficient and effective results for the segmentation of normal lungs, but are not suitable for the cases of diseased lungs or image artifacts.

### Advanced

This class includes algorithms that are more robust and do not have the inherent limitations of the simple category algorithms but are generally much more computationally expensive than the simple category algorithms. Approaches based on registration^7^, advanced thresholding with adaptive border matching^8^ and texture features^9^ fall within this category.

### Hybrid

This class includes approaches that utilize the approaches in category 2 only if the methods in category 1 fail to provide the perceived result on the basis of some heuristics (e.g. lung volume assumptions).

The Authors in^10^ have proposed an ensemble classification technique that is aided by clustering. In their approach they have utilized clustering for training dataset classification. For SVM training, the resulting nodule and non-nodules in the clustering step are used. Maeda *et al*.^11^. have proposed a technique that is a mix of ANN, Genetic Algorithm (GA) and SVM. They have opted to use temporal subtraction of consecutive CT scan images for the detection of candidate nodules. In the first phase, the candidate nodules’ features are computed that are later refined by utilizing rule-based feature analysis. They have utilized Principal Component Analysis (PCA) for feature space reduction. Later, The Artificial Neural network (ANN) is employed for classifying the nodules. They have deployed existing well-known techniques for the segmentation phase. The estimation of the center of the nodule is performed by using divergence of normalized gradient and nodule and vessel enhancement filtering is utilized for the segmentation of clusters of nodules. This is followed by the invariant, shape and regional descriptor calculations. Choi *et al*.^12^. have adopted a technique in which thresholding, contouring correction and morphological operation are used to extract the lung volume. They first utilize multiple thresholding scheme for extracting candidates from lung volume. This step is followed by the pruning of resultant candidates. The rules for pruning are defined on the premise of the type of features of the candidate nodules. Genetic Programming (GP) classifier is then trained and used for classifying the nodules and non-nodules. Choi *et al*.^13^. provide further insights and propose a hierarchical approach to the classification of nodules by means of SVM. In this study, the input image is first processed to obtain non-overlapped blocks and then those blocks are discarded which are non-informative. The features are extracted from the enhanced blocks and SVM was used for classification of candidate nodules. A new approach for lung nodule classification is presented in^14^. In this work, the input image is transformed to frequency domain using wavelet transform instead of using segmentation. After that gray level co-occurrence matrix (GLCM) was employed for extraction of texture features. Sheeraz *et al*.^15^. proposed a novel hybrid feature based method for nodule detection. The 3-dimensional and 2-dimensional statistical features are extracted from candidate nodules. The reported the sensitivity rate of 95.31%. In another interesting approach^16^, the optimal threshold value for segmentation was obtained using Gaussian approximation based differential evolution technique. For extraction of optimized features, they proposed a feature descriptor based on gradient intensity. The obtained accuracy and sensitivity were 98.7% and 97.5%. Naqi *et al*.^17^. presented nodule detection technique which is based on geometric fit in parametric form. The hybrid geometric feature was extracted for better representation which comprise on 2D as well as 3D information about nodules. They have achieved sensitivity rate of 95.6% on lung image database consortium images. Prewitt *et al*.^18^. have utilized the mode method technique for the selection of thresholds at the valleys on the histogram. Their proposed technique requires histogram data to be smoothed to automatically select, search for modes and place thresholds at the minimum between them. Their proposed technique is excessively dependent on the gray level histogram structure carrying peaks and valleys that are consonant with the image’s gray level subpopulation. The major problem with this approach is that the simple Heuristic search method is inadequate for finding the two peaks. Moreover, the bottom of the valley is difficult to find and calculate the exact threshold in case of flat valley.

Although the techniques discussed above obtained good results on normal lung images, however, the decrease in image quality may degraded their system performance which results in loss of important diagnostic information.

In this paper, we proposed a novel framework for lung segmentation that reduce the false positive error rate and improve accuracy rate for low contrast images and noisy images.

Major contribution of the proposed technique includes.

- The hierarchical block structure is used to preserved the image details such as nodules and blood vessels.
- Image contrast is enhanced in frequency domain and image details are preserved.
- Extraction of the most discriminative features from lung nodules.

The rest of the paper is organized as follows: Section 2 provides the description of the materials and methods. Experimental results are presented in Section 3. Conclusion and future directions are provided in Section 4.

## Material and Methods

The performance of accurate lung nodule detection is highly dependent on the image contrast enhancement and the accurate feature extraction. That is why the contrast enhancement and feature extraction step is considered as the most important steps. The aim of contrast enhancement is to improve the visual quality of an input image before feature extraction. In this paper, we present an effective contract enhancement technique that can not only improve the image contrast, but also preserve the brightness.

In order to enhance the image contrast, the input CT scan image is first converted into low-frequency (LF) component and high-frequency (HF) component using discrete cosine transform. Then Contrast limited adaptive histogram equalization (CLAHE) is employed to enhance the low-frequency component and the high-frequency component information remain unchanged. The reason is that most of the image noise is contained within high-frequency component.

**Figure 2:**
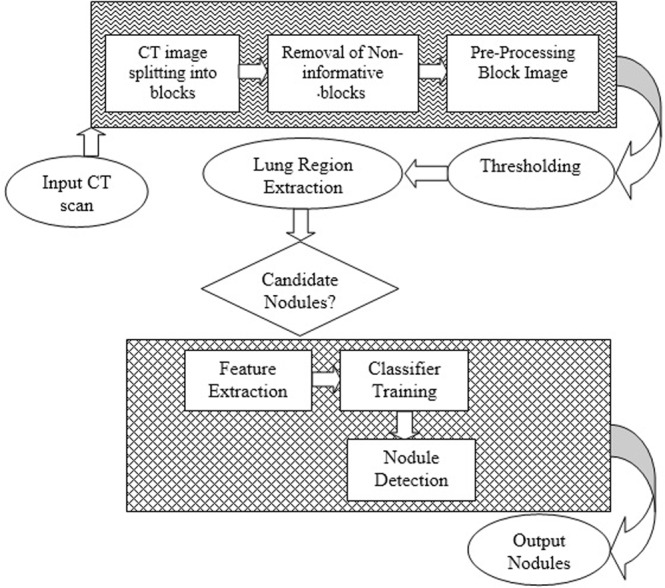
Proposed framework flow diagram.

After contrast enhancement, the image is divided into non-overlapping blocks and the non-informative blocks are filtered. In next step, thresholding is applied for extraction of lung region. In feature extraction step, weber local descriptor (WLD) is used to compute two components: differential excitation and orientation. These components describe and capture the texture information of an image. Finally, SVM classifier is trained and tested on the extracted features to classify nodules and non-nodules. The details of the proposed method are described in sub-sequent sections. Figure 2 illustrate the schematic diagram of our proposed method.

### Preprocessing

**Figure 3:**
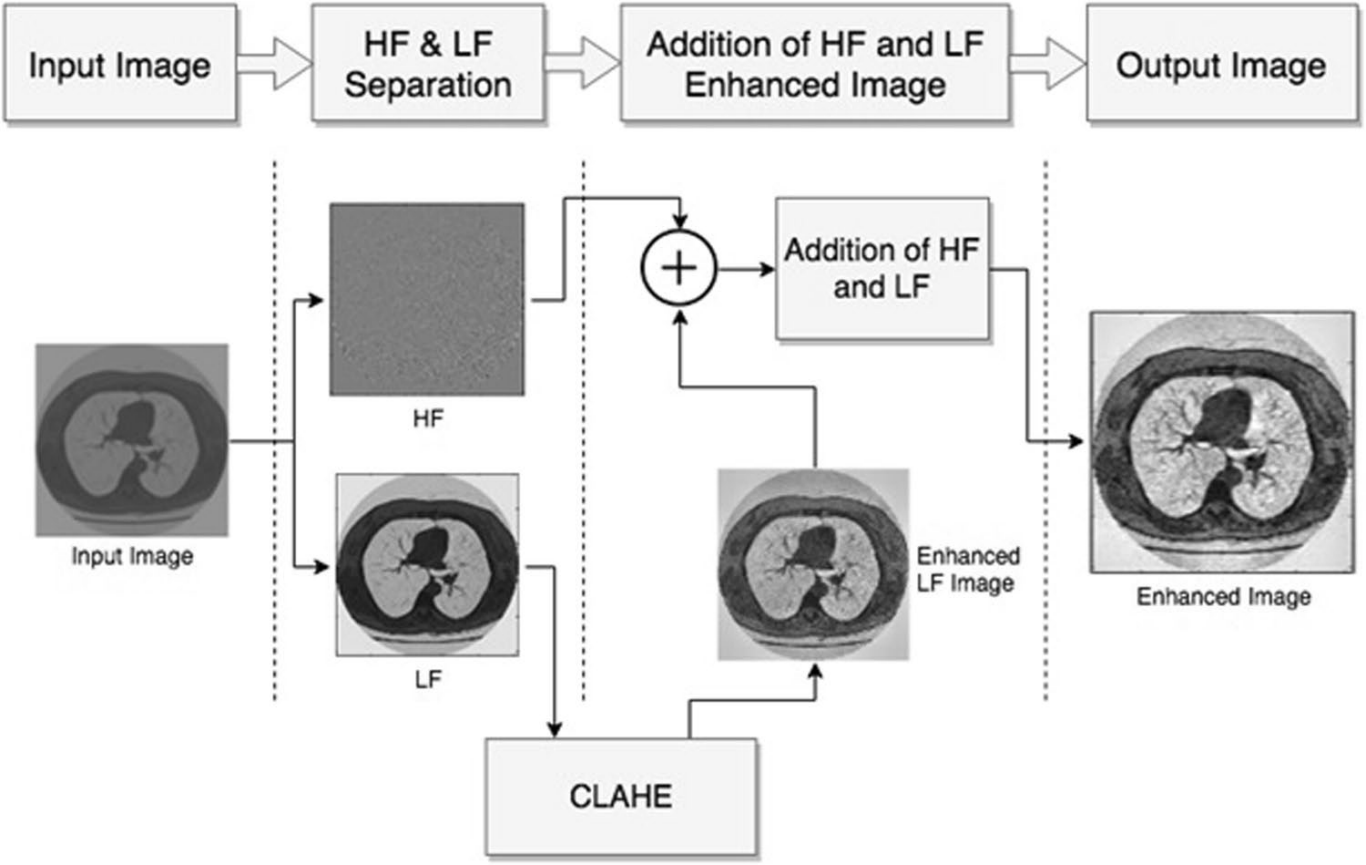
The flow diagram of pre-processing module.

In this paper, we introduce an efficient and simple framework to enhance contrast of the image without boosting noise levels in the compressed domain. Figure 3 illustrate the flow diagram of proposed pre-processed method.

Generally, spatial and frequency domain are used for image contrast enhancement. The detail of an image can be well obtained by transforming the image from spatial to frequency domain^19^. The focus of the spatial domain techniques is mainly on local information while frequency domain techniques explore the global information of an image. In frequency domain the image is converted into high and low-frequency component. The low-frequency component of the image contains the image detail while noise exists in the high frequency component. The good side of the frequency domain technique is that the image contrast can be enhanced without amplification of noise. In this paper we utilized discrete cosine transform to produce low and high frequency component of an input image.

We can compute the DCT of a input scan image *d*_*x,y*_ of size *M* × *N*, by using the expression as defined in Eq. 1. For all values of *u* = 0, 1, 2 …, *M−*1 and *v* = 0, 1, 2, …, *N −* 1 expression of Eq. 1 must be evaluated. Also, given *D*_*u,v*_, for *x* = 0, 1, 2…, *M* − 1 and *y* = 0, 1, 2,…, *N* − 1, *d*_*x,y*_ can be obtained by inverse DCT transform which is mentioned in Eq. 2. Note that both equations (1) and (2) consist of a two-dimensional pair of DCT, where *x* and *y* are spatial coordinates and, *u* and *v* refers to frequency variables^20^.

1 $${D}_{u,v}=\rho (u)\rho (v){\sum }_{x=0}^{M-1}{\sum }_{y=0}^{N-1}{d}_{x,y}\,\cos [\frac{(2x+1)u\pi }{2M}]\cos [\frac{(2y+1)v\pi }{2N}]$$

2 $${d}_{u,v}={\sum }_{u=0}^{M-1}{\sum }_{v=0}^{N-1}\rho (u)\rho (v){D}_{u,v}\,\cos [\frac{(2x+1)u\pi }{2M}]\cos [\frac{(2y+1)v\pi }{2N}]$$

$$\rho (u)=\{\begin{array}{l}\sqrt{\frac{1}{M}},u=0\,\\ \sqrt{\frac{2}{M},u=}1,2,3,\cdots ,M-1\end{array}$$

$$\rho (v)=\{\begin{array}{l}\sqrt{\frac{1}{N}},v=0\\ \sqrt{\frac{2}{N}},v=1,2,3,\cdots ,N-1\end{array}$$

$$\{\begin{array}{c}\begin{array}{c}\,u=0,1,2,\cdots ,M-1\\ v=0,1,2,\cdots ,N-1\end{array}\\ \begin{array}{c}x=0,1,2,\cdots ,M-1\\ y=0,1,2,\cdots ,N-1\end{array}\end{array}$$

The power spectrum *P*(*u, v*) of image *d*_*x,y*_ is defined in Eq. 3:

3 $$P(u,v)={|{D}_{u,v}|}^{2}$$

That is, the energy of the image is defined as the sum of squares of the DCT coefficients^21^.

Due to the large frequency values that DCT coefficients have at the origin, it’s generally referred to as the direct current (DC) element of the gamut whereas other coefficients are known as alternating current (AC) elements. The DC coefficients within the higher left corner show facts of lower frequencies, while the AC coefficients within the lower right corner relate to facts of upper frequencies. The fundamental characteristic of DCT is focusing in low-frequency components the foremost energy of a representative image. This means that the high-frequency component coefficients are nearly zero and considered negligible in maximum cases. Utmost data are included in the components of low-frequency of the spatial image, which symbolize a coarse or blurred version^22^.

The image’s low-frequency component is then enhanced with CLAHE. Instead of working on an entire image, the CLAHE decomposes the image into different regions and determines the number of histograms corresponding to each data region. In order to avoid over enhancement, CLAHE uses contrast limiting approach for each neighborhood point from which the transformation function is derived in a particular region. The CLAHE^23^ equation from which the new gray levels can be obtained as:

4 $$J=({j}_{max}-{j}_{min})\times P(f)+{j}_{min}$$

Where *j* is the new pixel value we want to generate, the maximum and minimum pixel values correspond to *j*_*max*_ and *j*_*min*_. *P*(*f*) is the distribution of cumulative probabilities.

**Figure 4:**
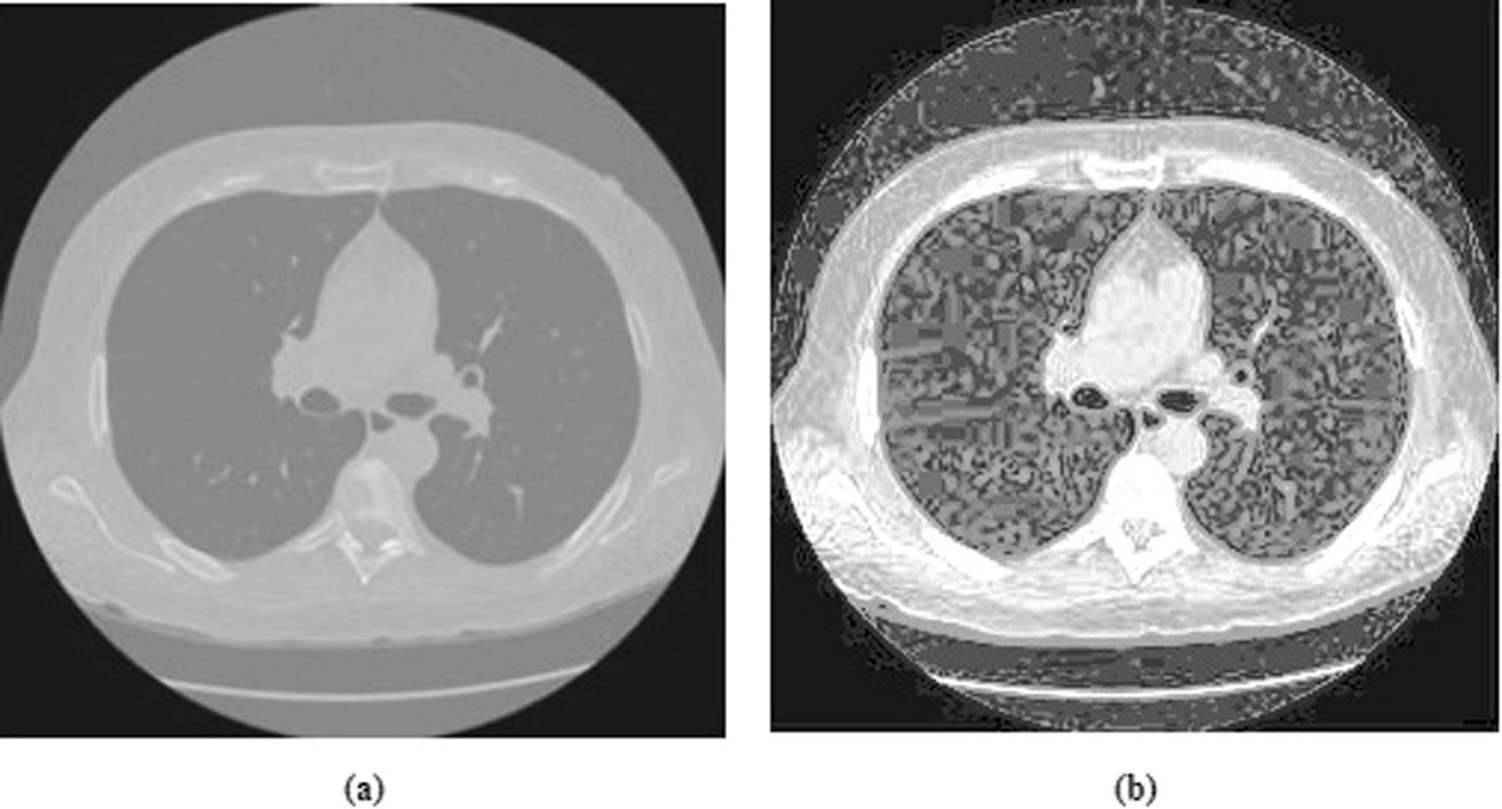
CT image contrast enhancement results. (**a**) Before contrast enhancement. (**b**) After contrast enhancement.

At the starting point of background and the ending point before fat, the display range of the image is expanded for the whole pixels range as illustrated in Fig. 3. Results of the contrast stretching are shown in Fig. 4.

### Thresholding

Thresholding technique is the simple and efficient way for segmentation of the images. The segmentation is based upon directly on pixel intensities. Due to the overlap between the background intensities and some sections of ROI, simple thresholding may not be suitable for extraction of lung region^24^. The background region of the respective images is discarded to overcome this problem. The proposed segmentation technique used a combination of optimal thresholding based on differential evolution^25^ and corner-seeded region growing.

When background of the scan image is removed, optimal thresholding based on differential evolution is employed to determine the boundary of lung region and lung area extraction. The initial threshold of −950 HU is applied, as the majority of the lung region ranges from −950 HU to −500 HU. It is an iterative process in which each iteration recalculates the threshold.

In an image, histogram is used to obtain the probability distribution for various gray levels. This distribution of probabilities is calculated by Eq. 5 in the first place.

5 $$p(x)={\sum }_{i=1}^{K}{P}_{i}\cdot {p}_{i}(x)\,=\,{\sum }_{i=1}^{K}\frac{{P}_{i}}{\sqrt{2\pi {\sigma }_{i}}}exp[\frac{-{(x-{\mu }_{i})}^{2}}{2{\sigma }_{i}^{2}}]$$

*K* represents the total number of categories in the scan image. *P*_*i*_ and *pi*(*x*) are the distribution functions of probability and probability in category *i*. *M*_*i*_ represent the mean and *σ*_*i*_ is standard deviation. For both different categories, the overall probability error is reduced by Eq. 6. This is used to calculate the optimal threshold.

6 $$e({T}_{i})={P}_{i}{\int }_{-\infty }^{{T}_{i}}{p}_{i}(x)dx+{P}_{i+1}{\int }_{{T}_{i}}^{\infty }{P}_{i+1}(x)dx$$

This error relates to the *T*_*i*_ threshold. The overall error is then computed in accordance with Eq. 7.

7 $$E(T)={\sum }_{i=1}^{K-1}e({T}_{i})$$

**Figure 5:**
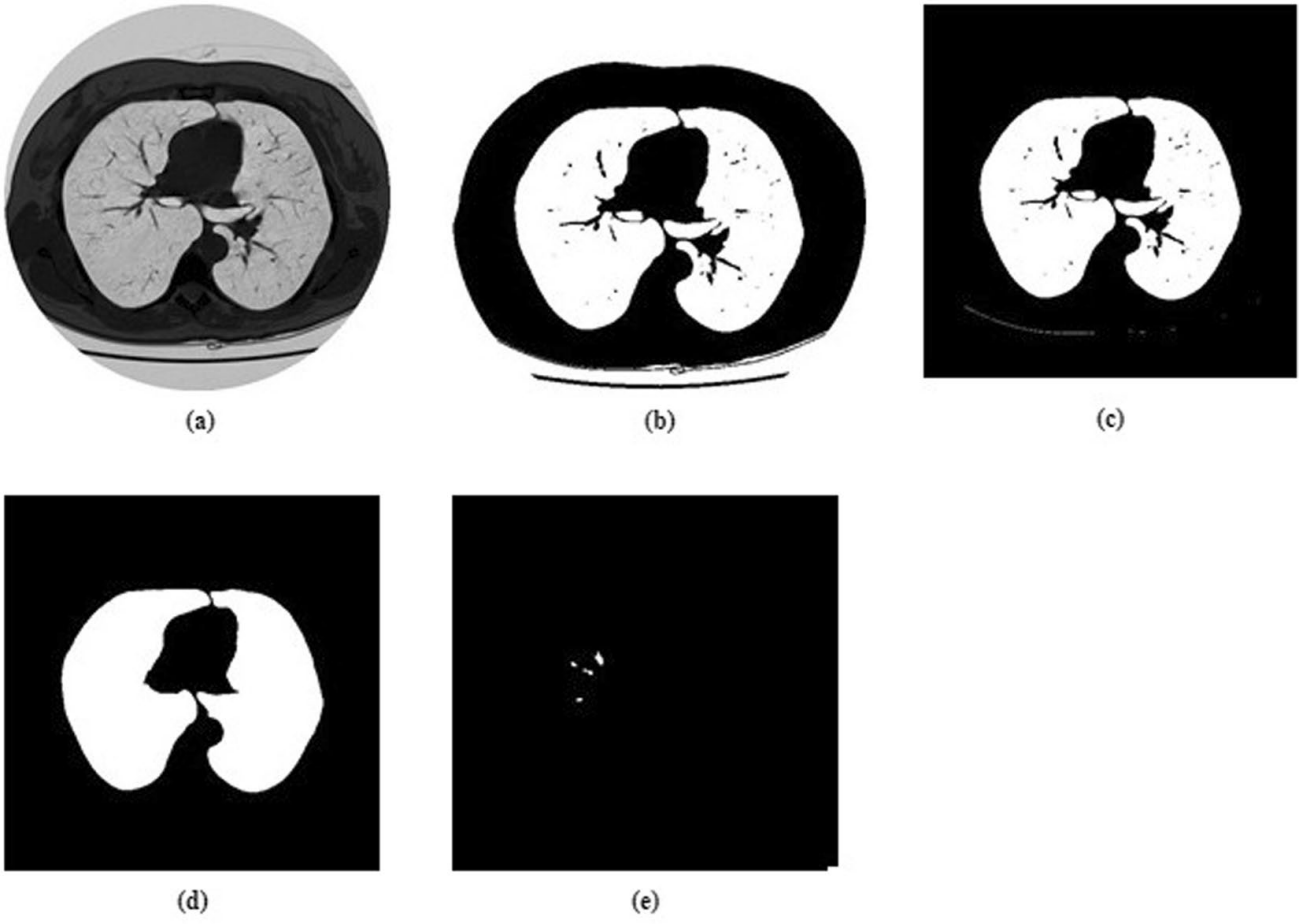
Lungs segmentation and candidate nodule detection. (**a**) Original CT scan slice. (**b**) Threshold CT scan slice image. (**c**) Background removed slice image. (**d**) Hole filled CT scan slice image. (**e**) Image of candidate nodules.

A threshold image containing the lung mask is now generated. Figure 5(b) illustrates the CT scan threshold image.

### Background Removal

By simply applying the image threshold, we cannot get a whole part of the lungs from the background. From Fig. 4, it is clearly evident that the gray levels of image background and of the lungs are highly similar. Therefore, a mechanism is needed to eliminate the entire background. Initially, a background removal operator is used to remove the background^26^. This operator moves along the four directions beginning at four corners of the target image. It identifies the image background pixels by using the range values of grey levels and then removes the particular pixels till the pixels surpass the range values or length of rows or columns. Further, the image is traversed from top to bottom at middle. The resulting image consists only of the chest and the lungs segments.

### Candidate Nodule Extraction

The result of the preprocessing step is the 3D lung mask, which is subsequently used to extract lung volume from the original Lung CT. The lung volume extracted consists of nodules and vessels. Due to their intrinsic density variations, nodules and vessels tend to be denser than the lung.

In order to extract the ROIs, threshold is computed using the median slice which is the best available thresholding technique. It should be noted that, since vessels and nodules have different levels of density, multiple threshold values must be calculated based on nodule type.

### Candidate Nodule Pruning

The resulting ROIs are nodules and vessels. The general diameter range of the nodule is between 3 mm and 30 mm. ROIs <3 mm diameter are therefore excluded as noise and, ROIs >30 mm diameter are pruned as lesions or vessels. To detect the vessels in the ROIs, the elongation property is used.

### Feature Extraction

For nodule detection and classification, relevant features play important role. In this study, we applied WLD to extract the local features.

As proposed by Chen *et al*.^27^, local descriptor technique called WLD is used to classify texture and face detection. This technique comprised of two processes: (a) the differential excitation, which describes a central pixel’s relative intensity differences from its neighbors and (b) orientation, which describes the central pixel’s gradient orientation. These two processes would produce complementary information for the description of local texture. From the literature^27^, Weber significance and orientation are described as follows:

Weber magnitude:

8 $${\varepsilon }_{m}({x}_{c})=\arctan (\alpha {\sum }^{}\begin{array}{c}p-1\\ i=0\end{array}\frac{{x}_{1}-{x}_{c}}{{x}_{c}})$$

Where the *arctan* function is applied to forestall the output from being large and therefore might partly subdue the noise side-effect. *x*_*c*_ is the center pixel, *xi* = 0, 1, …, p−1 is the adjacent pixels and $\frac{{x}_{i}-{x}_{c}}{{x}_{c}}$ is the differential excitation between *x*_*c*_ and *x*_*i*_, *p* is the count of neighbors and *a* is an attribute to adjust the differences in intensity between adjacent pixels. If ε_*m*_(*x*_*c*_) is zero or close to zero, it is mainly flat area^28^.

The orientation factor can be described as the ratio of the horizontal direction change to the current pixel’s vertical direction. Sobel operator is used to obtain gradient orientation and can be calculated as;

9 $${\varepsilon }_{o}({x}_{c})=\arctan \frac{{x}_{1}-{x}_{5}}{{x}_{3}-{x}_{7}}$$

where *x*_1_ − *x*_2_ and *x*_3_ − *x*_7_ shows differences in the intensity *x* and *y* direction respectively.

### Support Vector Machine (SVM) for Nodule and Non-Nodule Classification

The classification of patterns is described as the task of categorizing any object in a specified class type. Vapnik developed the SVM to solve the problems of classification. Cortes and Vapnik developed the present version of SVM classifier for regression at AT&T laboratories in 1995^29^. For binary classification problems that have only two different classes, the theoretical characteristics of SVM are typically defined.

SVM’s basic idea is to build a hyperplane that maximizes the margin between positive and negative examples. The hyperplane is determined by the supporting vectors closest to the surface of the decision. The decision surface is determined by the internal product of the training data, which allows us to map the input vectors to a higher-dimensional internal product area called the feature space. The input function vector is displayed in the form N*M matrix below.

10 $$R={\{{v}_{(n)}\}}_{n-1}^{N}$$

The total number of feature vectors is indicated here by *N* and *M* dimensional feature vector is represented by *v*. The SVM finds the hyper plane of higher dimensional space in the training process and separates the nodules from non-nodules.

## Experimental Results and Discussion

In this section, we evaluate and analyze the performance of the proposed method on LIDC image dataset of chest CT images^30^. As LIDC database contain images that were collected from various institutes, the spatial resolution and X-ray image parameters varied (slice intervals, 0.625–3.0 mm; resolution in the plane, 0.488–0.946 mm; tube voltage, 120–140 kV; and tube current, 40–499 mA). In this work, we focus our attention on nodules with a diameter of 5–20 mm, which were identified as a nodule by at least one doctor in four. By using the LIDC database, we considered 84 cases among which included 103 nodules in total.

### Quantitative metrics for evaluation

The proposed diagnostic system is evaluated in terms of performance by means of well-known metrics, including sensitivity, accuracy, and specificity. These measurements are calculated using True Positive (TP), True Negative (TN), False Positive (FP) and False Negative (FN). Where TP is the likelihood that a cancer patient has cancer. The FP is the probability that cancer is found to be the detection value of a healthy person. The TN is the likelihood that the cancer patient is healthy. The FN is explained as a healthy person is having cancer.

### Accuracy

Accuracy is the measure of the classification scheme’s overall effectiveness/usefulness. It can be calculated using the following equation.

11 $$Accuracy=\frac{TP+TN}{TP+FP+TN+FN}\times 100$$

### Sensitivity

Sensitivity is termed as the capability of a classifier to detect positive class patterns. The following equation can be used to obtain it.

12 $$Sensitivity=\frac{TP}{TP+FP}$$

### Specificity

Specificity is termed as the capability of a classifier to detect negative class patterns. Specificity can be obtained from the following equation.

13 $$Specificity=\frac{TN}{TN+FP}$$

**Figure 6:**
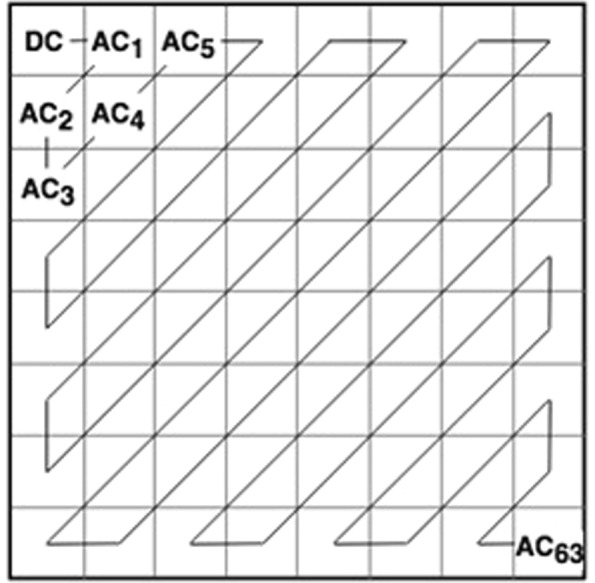
A Zig-zag mechanism of DCT.

To enhance the contrast of the images along with preserving the image detail, we first preprocessing all the image for possible contrast enhancement. The image is divided into a block of 8 × 8 size. Each block is then converted into frequency domain via DCT. The upper left corner of DCT that is *d*(0, 0) is the *DC* coefficient and the other sixty-three elements are *AC* coefficients as shown in Fig. 6. The coefficients are sorted from upper left to lower right corner in order of increasing spatial frequencies.

**Figure 7:**
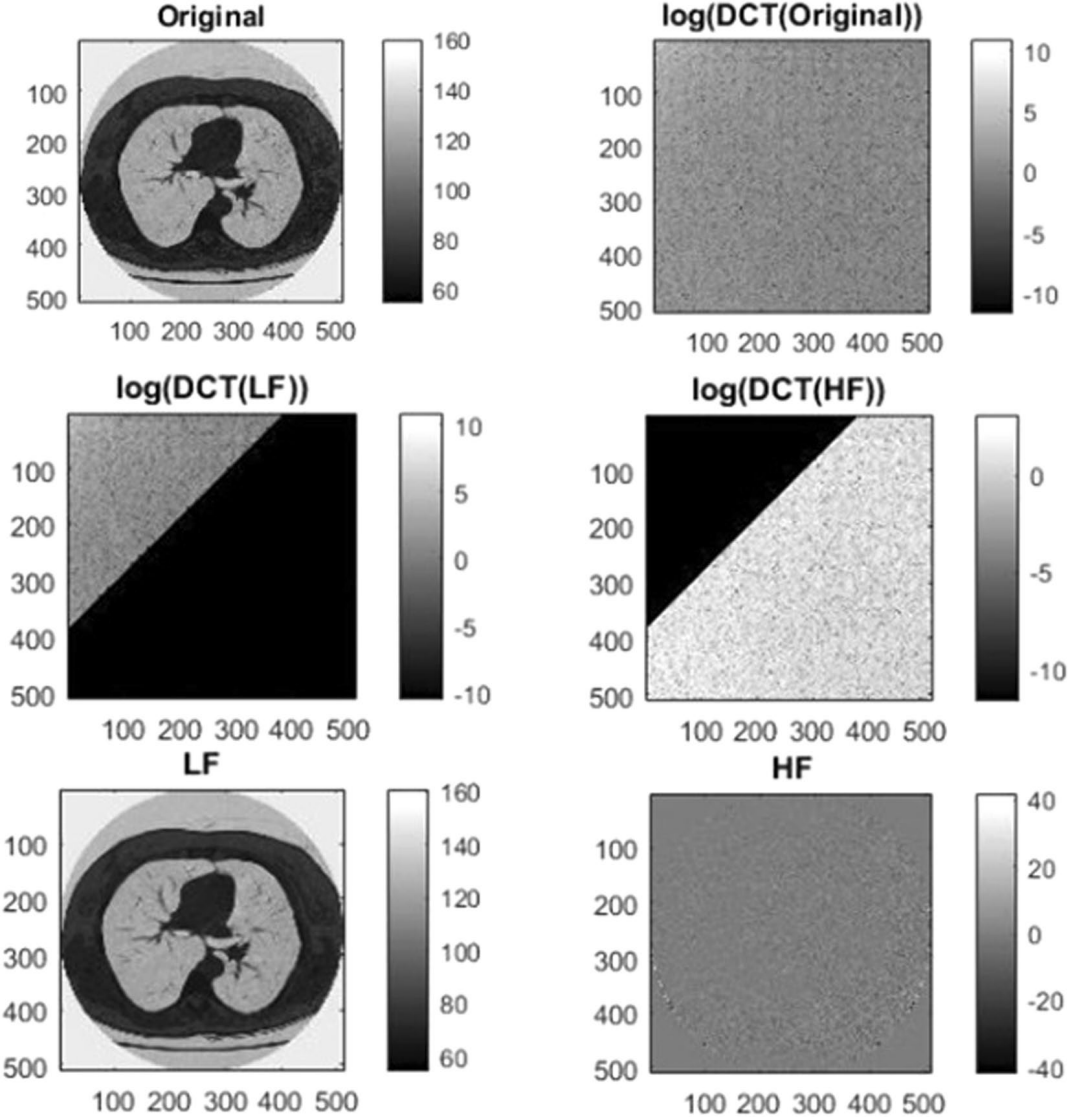
A sample images of LF and HF in frequency domain.

Figure 7 illustrate the input image conversion into low-frequency (DC) and high-frequency (AC) component using DCT. As shown in the figure, the LF component contain most of the image detail and HF component is mostly noisy.

**Figure 8:**
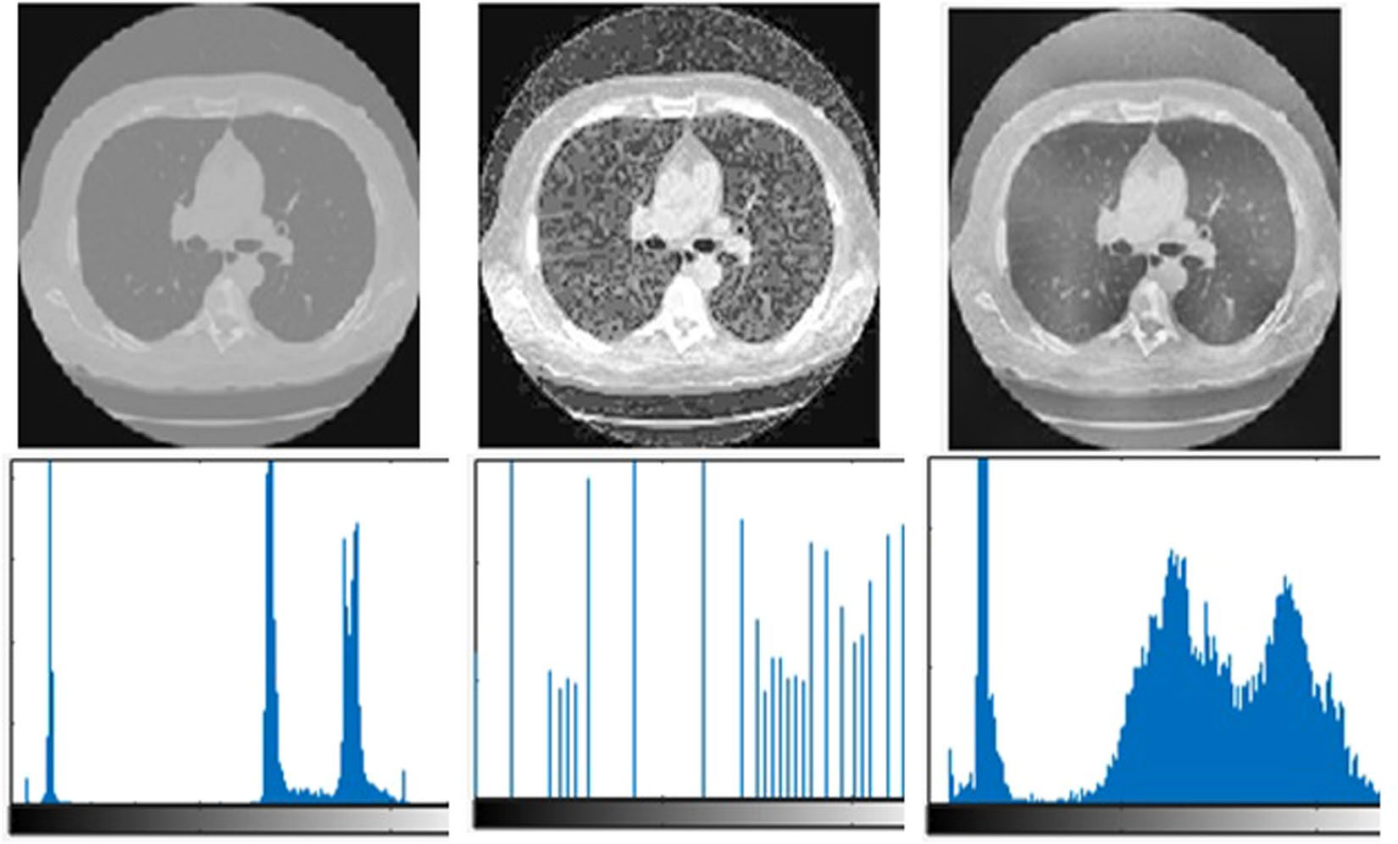
Visual results comparison with histogram equalization.

To avoid the over-enhancement and preserving the image details, we only enhanced the low-frequency component using CLAHE and the high-frequency component is kept unchanged. The visual results of the proposed CLAHE-DCT method is compared with classical histogram equalization method (see Fig. 8).

As shown in Fig. 8, the histogram equalization method does not improve the image detail information and it tends to over-enhanced the input image. In contrast, our proposed method preserved the edge and texture details also sufficiently improve the image brightness.

In next step, the background is removed from the images via operator used in^26^. For segmentation, we used differential evolution-based optimal thresholding^25^. This is because the simple thresholding is failed to achieve good performance. After candidate nodule extraction, feature extraction from the candidate nodule is another important step.

To perform the feature extraction step, we first compute the corresponding orientation and differential excitation component of an image. Each image of differential excitation and orientation is then divided into *N* non-overlapped blocks *R*_1_, *R*_2_, *R*_3,_ …. *R*_*N*_. WLD histogram *H*_*n*_(*n* = 1, 2, 3, …N) is then constructed for each block of differential excitation and orientation image. In next step, the WLD histograms of each block is integrating to construct enhanced features vector which can be used for classification.

$$H=\{{H}_{n}\}where\,n=1,2,3,\ldots \ldots .,N$$

**Figure 9:**
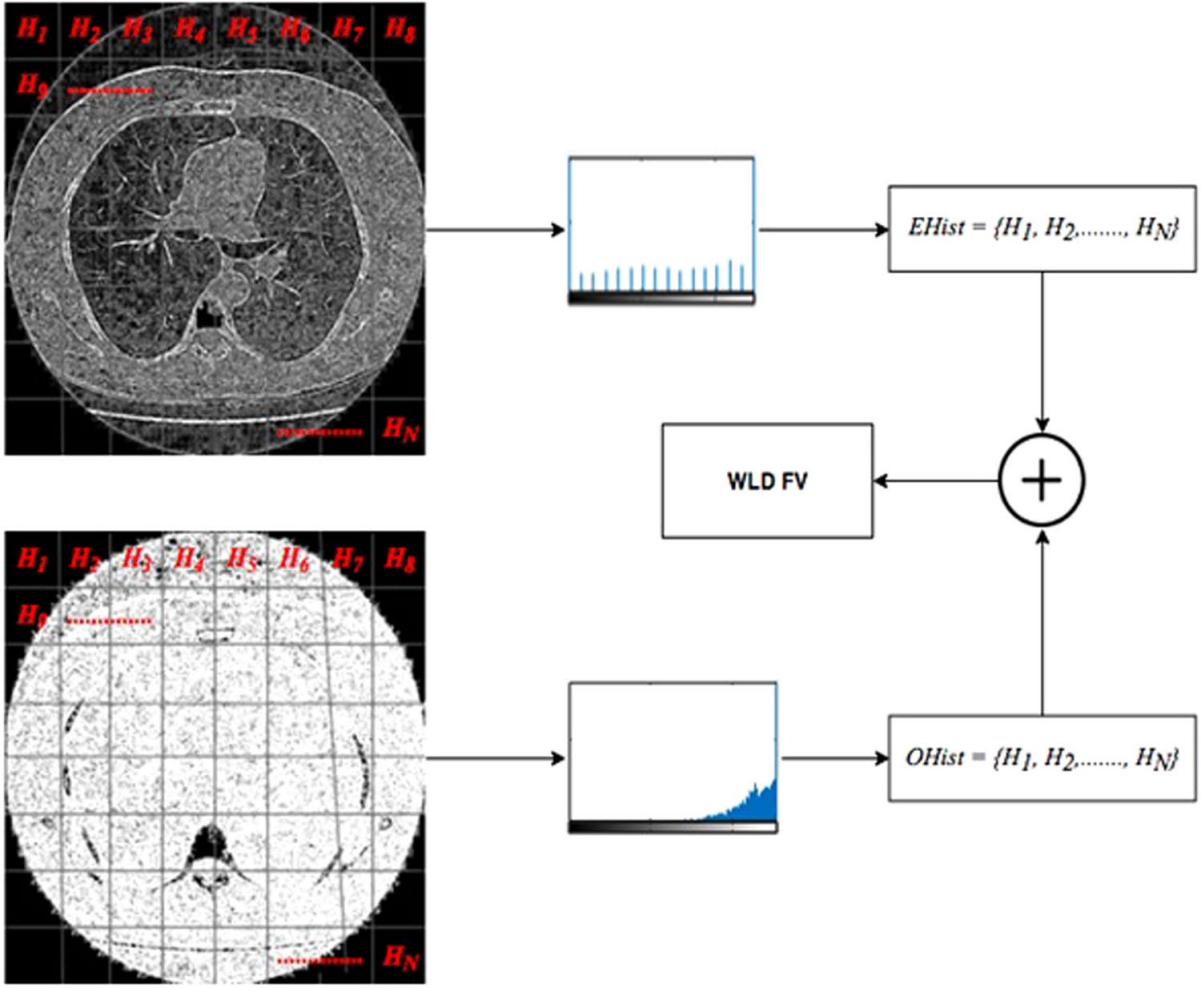
Feature vector generation from WLD images.

In last step, the feature vectors of differential excitation *EHist* and orientation component *OHist* are fused together to generate more robust representation of input image. Figure 9 illustrate the feature extraction process.

The feature vectors of different sizes FS-10, FS-12, FS-14, FS-16 and FS-18 are extracted using feature extraction step.

For training and testing purpose, we divided the dataset in the following manner:

- 70% and 30% training and testing ratio
- 50% and 50% training and testing ratio
- 30% and 70% training and testing ratio

**Figure 10:**
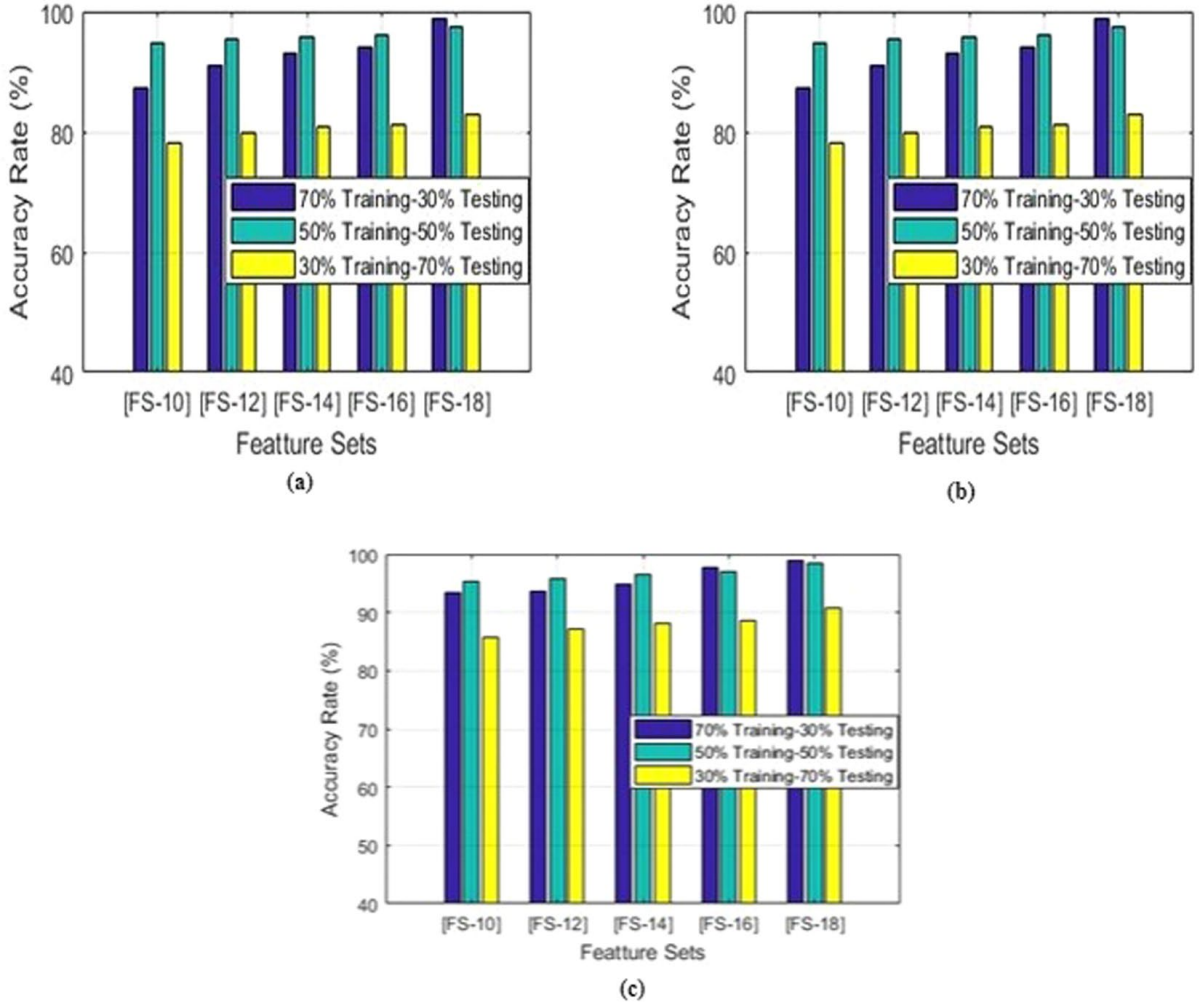
SVM classifier summarized results using different training and testing ratio and feature sets. (**a**) Sensitivity. (**b**) Spec. (**c**) Accuracy.

SVM results with different training to testing ratio are shown in Fig. 10.

### Sensitivity and specificity of FS-14 for 70-30 training and testing ratio

In order to perform the experiment using 70–30% training to testing ratio, we have divided the dataset into training to testing samples in which 1100 samples are reserved for training and 472 samples are used for testing. The testing set contains 236 non-nodules and 236 nodules. For 50–50% training to testing ratio, we used 393 samples as a training set and 393 samples as a testing set. Similarly, in case of 30–70% training to testing ratio, the total number of training and testing samples 472 and 1100. The results of correctly classified and miss-classified nodules/non-nodules are shown in Table 1.

**Table 1:** Classification of Nodule and Non-nodule using 70–30%, 50–50% and 30–70% ratio.

Training to Testing Ratio (%)	TP	FP	FN	TN	Total Nodules	Total Non-Nodules
70–30	233	2	3	234	236	236
50–50	383	4	10	389	393	393
30–70	456	8	94	542	550	550

True Positive -> TP, False Positive -> FP, False Negative -> FN, True Negative -> 234.

We have obtained 98.73% sensitivity, 99.15% specificity and 98.94% accuracy rate in case of 70–30% training to testing ratio. The results of 50–50% training to testing ratio is 97.45% sensitivity, 98.98% specificity and 98.35% accuracy rate. We have observed that in case of 30–70% training to testing ratio the performance is reduced that is 82.9% sensitivity, 98.54% specificity and 90.72% accuracy rate.

### Performance evaluation using K-fold cross validation

**Figure 11:**
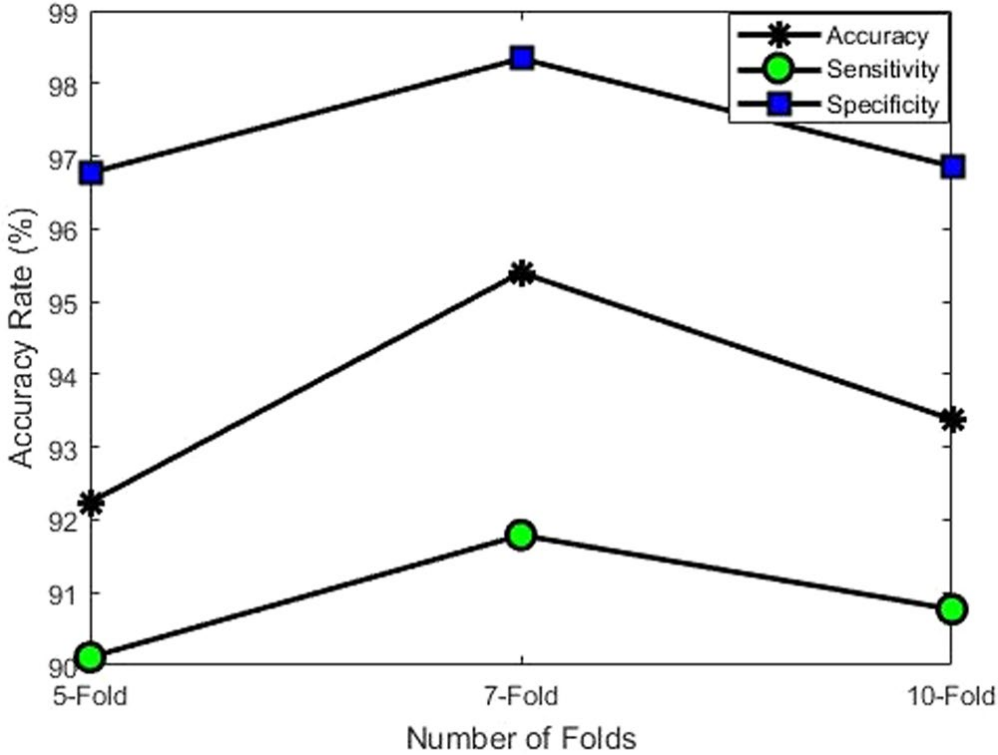
Performance of proposed framework with different folds.

We have also evaluated the performance of SVM by k-fold cross validation. For k-fold cross validation we set the value of *k* to 5,7 and 10. The performance of the SVM classifier on k-folds are shown in Fig. 11. As shown in the Fig. 11, 7-fold cross validation provides more better results as compared to 5 and 10-folds. We have also observed that there is a small difference between the performance of different k-fold which shows the robustness of the proposed method.

**Figure 12:**
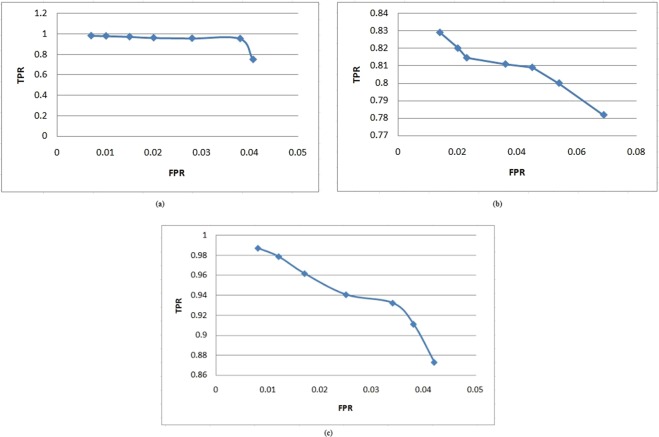
Comparison of TPR and FPR for different training to testing ration. (**a**) TPR and FPR for 50–50 training to testing ratio. (**b**) TPR and FPR for 30–70 training to testing ratio. (**c**) TPR and FPR for 70–30 training to testing ratio.

In order to represents the comparison in better way, we have also plotted curves for different training to testing ratio which illustrate that how correctly SVM classifier make a difference between nodules and non-nodules. The curve of true positive rate (TPR) against false positive rate (FPR) obtained for SVM is illustrated in Fig. 12. It is worth to note that although for three type of training to testing ratio we have obtained stable results using SVM, however, true positive rate for 50–50% training to testing ratio is slightly higher as compared to 70–30% and 30–70% training to testing ratio.

**Figure 13:**
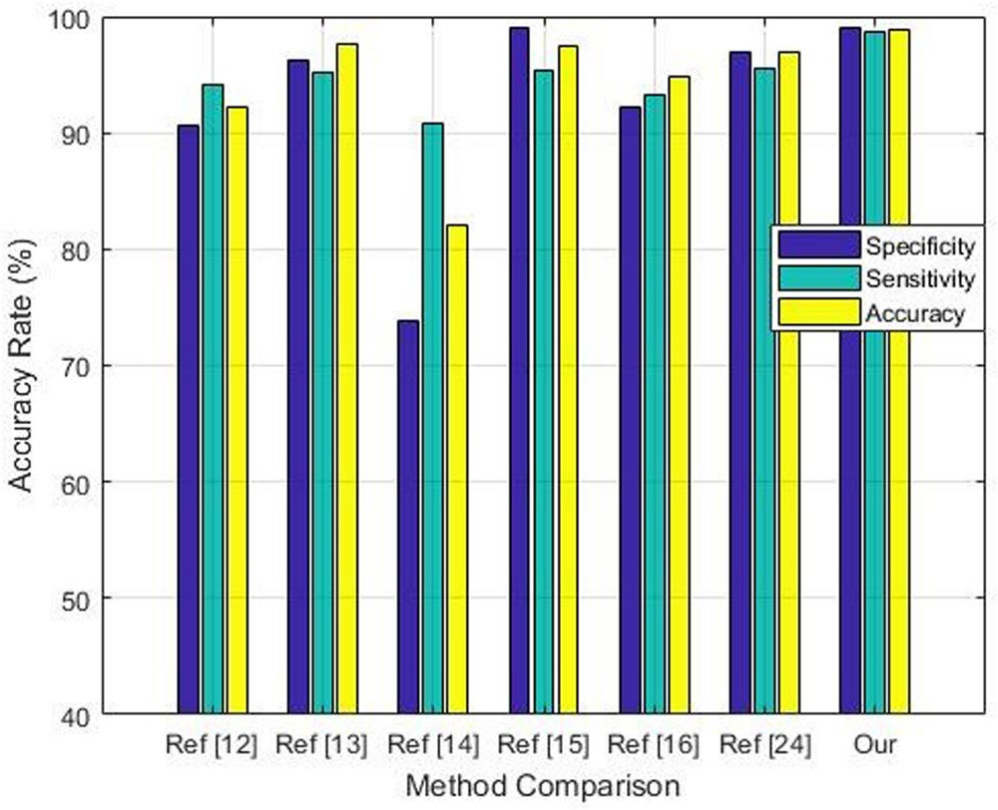
Performance comparison of proposed framework with other techniques.

One of the important factors in performance analysis is to compare the results with existing methods reported in literature. There are many methods reported in literature^31–34^ (with different domains) who follow the same experimental protocols. Such comparison is mandatory to evaluate the importance of diagnostic method. However, due to difference in experimental protocols this type of comparison is also very challenging. These include performance metrics, nodule size and type of dataset used. We have selected those methods which used accuracy, sensitivity and specificity as a performance metrics and also performed experiments on LIDC dataset. A brief comparison of proposed method and methods reported in literature is provided in Fig. 13. As shown in Fig. 13, our proposed method reported 99.15% specificity, 98.73% sensitivity and 98.94% accuracy which shows improvement as compared to performance of existing method.

## Conclusion

In this paper, a novel and effective pulmonary nodule detection framework is proposed. In the initial phase, the contrast of the images is enhanced that increases the robustness for segmenting images with varying contrasts. Transformation from spatial domain to frequency domain is performed using DCT which reveals those features that are difficult to detect in the original spatial domain. Most CAD systems have a common weakness that their system fail to perform well on low contrast medical images. In this study, we have proposed an effective framework for image contrast enhancement in frequency domain without boosting the noise. The proposed method has reduced false positives significantly in nodule candidates by using the most discriminative texture features. The empirical results provide the evidence that the proposed method can efficiently classify nodules and non-nodules. In the future, we are planning to use evolutionary algorithms in order to search for optimal features. We would also like to ensemble different classifiers for performance improvement.
